# The unravelling of the complex pattern of tyrosinase inhibition

**DOI:** 10.1038/srep34993

**Published:** 2016-10-11

**Authors:** Batel Deri, Margarita Kanteev, Mor Goldfeder, Daniel Lecina, Victor Guallar, Noam Adir, Ayelet Fishman

**Affiliations:** 1 Department of Biotechnology and Food Engineering, Technion-Israel Institute of Technology, Haifa, 3200003, Israel; 2 Joint BSC-CRG-IRB Research Program in Computational Biology, Barcelona Supercomputing Center, Jordi Girona 29, 08034 Barcelona, Spain; 3 Institució Catalana de Recerca i Estudis Avançats (ICREA), Passeig Lluís Companys 23, 08010 Barcelona, Spain; 4 Schulich Faculty of Chemistry, Technion-Israel Institute of Technology, Haifa, 3200003, Israel

## Abstract

Tyrosinases are responsible for melanin formation in all life domains. Tyrosinase inhibitors are used for the prevention of severe skin diseases, in skin-whitening creams and to avoid fruit browning, however continued use of many such inhibitors is considered unsafe. In this study we provide conclusive evidence of the inhibition mechanism of two well studied tyrosinase inhibitors, KA (kojic acid) and HQ (hydroquinone), which are extensively used in hyperpigmentation treatment. KA is reported in the literature with contradicting inhibition mechanisms, while HQ is described as both a tyrosinase inhibitor and a substrate. By visualization of KA and HQ in the active site of TyrBm crystals, together with molecular modeling, binding constant analysis and kinetic experiments, we have elucidated their mechanisms of inhibition, which was ambiguous for both inhibitors. We confirm that while KA acts as a mixed inhibitor, HQ can act both as a TyrBm substrate and as an inhibitor.

Tyrosinases belong to the type 3 copper-containing protein family together with hemocynanins that serve as oxygen carriers^1^,^2^, and catechol oxidases that are strict diphenolases^3^,^4^. The two copper ions in the conserved active site, CuA and CuB, are coordinated by six histidine residues^5^,^6^,^7^. Tyrosinases hydroxylate monophenols to form *ortho*-diphenols (monophenolase activity) and subsequently oxidize the *o*-diphenols to *o*-quinones (diphenolase activity). Melanin is formed rapidly by the spontaneous polymerization of the quinones^5^,^8^. Monophenols can react only with the *oxy* state of tyrosinase, which represents about 15% of the enzyme molecules in solution^9^. In the presence of *o*-diphenols such as L-dopa (L-3,4-dihydroxyphenylalanine), both the *oxy* and *met* forms react enabling the production of *o*-quinones^4^,^9^.

Disorder in melanin formation has been found to cause a variety of skin diseases in humans such as hyperpigmentation, lentigo, vitiligo and skin cancer^10^. Furthermore, appearance of brown pigments in fruits and vegetables due to tyrosinase activity is a leading cause for postharvest losses^9^. Therefore, tyrosinase inhibitors are highly warranted by the pharmaceutical, cosmetics and food industries^11^,^12^,^13^,^14^,^15^.

Kojic acid (KA), a fungal metabolite, is the most widely used skin-whitening agent with possible side effects being dermatitis, sensitization and erythema^9^,^16^. Animal experiments suggested possible tumor promotion and weak carcinogenicity, and thus concentrations of 1% are recommended for safe human use^17^. Numerous contradicting mechanisms are described in the literature for KA as either a competitive or mixed inhibitor for mushroom tyrosinase^18^,^19^,^20^, possibly by chelating copper in the active site^18^,^20^,^21^,^22^. Previously, KA was found bound at the entrance to the active site of TyrBm (tyrosinase from *Bacillus megaterium*), suggesting one significant intermediate binding site. However, the full mechanism of KA inhibition still remains unclear^22^.

Hydroquinone (HQ), another well-studied whitening agent, has been used clinically in leading cosmetic hyperpigmentation treatment^23^, however, it was also found to cause serious problems by generating reactive oxygen species leading to oxidative damage of lipids and permanent loss of melanocytes. Subsequently, HQ has been banned for the general use by the European Committee and can be prescribed by dermatologists only^13^,^16^. Previous studies suggested that HQ is a competitive inhibitor of tyrosinase^24^,^25^, while others demonstrated the potency of HQ as a tyrosinase substrate^26^,^27^. Garcia-Canovas and co-workers suggested that the enzymatic activity is not evident since HQ is not able to transform *met*-tyrosinase to the *oxy*-form^26^, and that the transformation may be substantial by addition of an *o*-diphenol or H_2_O_2_^26^,^28^. However, to date, no structural data is available in order to elucidate the orientation of HQ in the active site of tyrosinase.

Most mechanistic studies on tyrosinase inhibitors use KA or HQ as the comparative benchmark compound. Therefore, in depth analysis of their mechanism and inhibition mode are crucial for further development of potent inhibitors.

In this study, we elucidate the inhibition mechanism of these inhibitors by crystal structure determination of TyrBm with bound KA and HQ in the active site, along with biochemical characterizations, binding constants determination and molecular modeling.

## Results

### Inhibition mode of TyrBm activity

**Figure 1:**
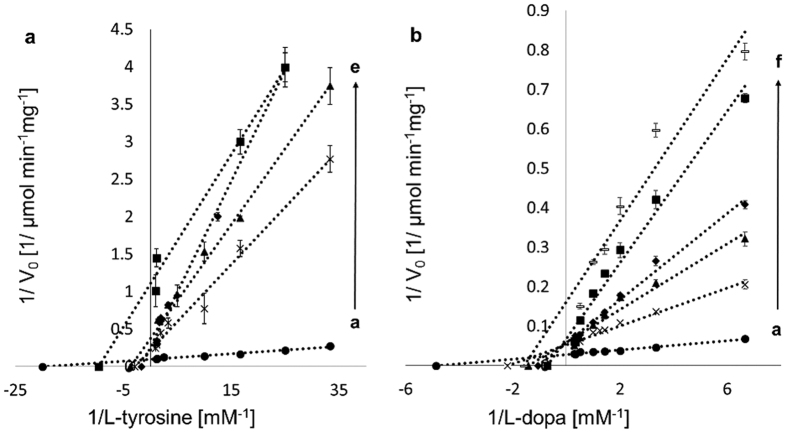
Lineweaver–Burk plots for the inhibition of TyrBm by KA. (**a**) L-tyrosine (0.03–1.4 mM) in the presence of KA concentrations (mM): (**a** ●) 0, (**b**×) 0.025, (**c** ▲) 0.05, (**d** ♦) 0.075, (**e** ■) 0.1 and (**b**) L-dopa (0.15–2.0 mM) in the presence of KA concentrations (mM): (**a** ●) 0, (**b**×) 0.025, (**c** ▲) 0.04, (**d** ♦) 0.05, (**e** ■) 0.075, (**f** -) 0.1. All measurements were performed in heptaplicates.

**Figure 2:**
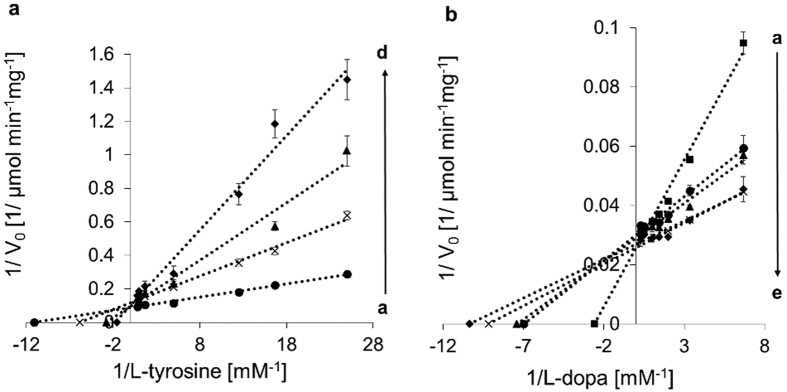
Lineweaver–Burk plots for the inhibition of TyrBm by HQ. (**a**) L-tyrosine (0.03–1.4 mM) in the presence of HQ concentrations (mM): (**a** ●) 0, (**b**×) 0.025, (**c** ▲) 0.075, (**d** ♦) 0.5 and (**b**) L-dopa (0.15–2.0 mM) in the presence of HQ concentrations (mM): (**a** ■) 0, (**b** ●) 0.025, (**c** ▲) 0.05, (**d**×) 0.075, (**e** ♦) 0.1. All measurements were performed in heptaplicates.

The most widely used and effective tyrosinase inhibitors, HQ and KA^9^,^29^, were tested for their inhibitory effect on TyrBm. Overall, our results clearly show that KA and HQ have different inhibition modes on TyrBm monophenolase (L-tyrosine) and diphenolase (L-dopa) activities. While KA displays a mixed inhibition mode on both activities (Fig. 1 and Table 1), HQ is a competitive inhibitor of monophenols, and shows no inhibition of diphenols (Fig. 2 and Table 1), in contrast to previous reports that define HQ as a competitive inhibitor for both activities^24^,^25^. Our kinetic study showed no inhibition by HQ in the presence of L-dopa since with rising concentrations of HQ, *K*_m_ values decreased (Fig. 2b and Supplementary Fig. S1a).

The IC_50_ values representing inhibitor concentrations in which TyrBm activity was reduced by 50% were obtained from dose-response curves (Supplementary Fig. S2). The IC_50_ values for KA and HQ on the monophenol were 26.8 and 32.0 μM respectively, while the IC_50_ value for KA on the diphenol was 52 μM (Table 1). Similar results were obtained in previous reports for mushroom tyrosinase, with values for KA inhibition on monophenols and diphenols of 5.7 and 30.1 μM, respectively^30^,^31^, and the value for HQ inhibition on monophenols of 33.5 μM^32^. Since we observed that HQ does not inhibit tyrosinase in the presence of L-dopa, the value of IC_50_ was not determined. García-Canovas and co-workers suggested that HQ is a tyrosinase substrate and not an inhibitor although activity is not evident under conventional conditions since HQ cannot transform *met*-tyrosinase into *oxy*-tyrosinase^26^. However, in the presence of an *o*-diphenol (e.g. L-dopa) or H_2_O_2_, *oxy*-tyrosinase is generated and HQ becomes a substrate which is hydroxylated to 2-hydroxyhydroquinone and subsequently to 2-hydroxy-*p*-benzoquinone (HPB) that can be measured spectrophotometrically^26^. Our spectrophotometric measurements confirm their results, since with the addition of H_2_O_2_ or L-dopa, product formation by TyrBm increased in the presence of HQ (Supplementary Fig. S1). Furthermore, the same trend was observed by direct measurement of TyrBm activity through recording of the oxygen consumption during the reaction (Supplementary Fig. S3). The activity of TyrBm in the presence of L-dopa and HQ was 8% higher than with L-dopa alone for oxygen consumption measurements, and 17% higher as determined by absorbance readings.

In addition, when an activity test was performed for several hours with HQ as a sole substrate in comparison to a control without enzyme, low activity was visually observed, even without the addition of H_2_O_2_ or L-dopa, and a light brown color was detected after 3 hours of incubation (the control remained colorless). Moreover, the low inhibitory effect of HQ was also evident in another kinetic study with mushroom tyrosinase that exhibited an IC_50_ value 80-fold higher when L-dopa was used, in comparison to L-tyrosine^24^. Since HQ requires a reducing agent in order to become a TyrBm substrate, the kinetic parameters of the monophenolase activity were determined in the presence of H_2_O_2_. The *K*_m_ and *V*_max_ values were 0.27 mM and 19 μmole min^−1^ mg^−1^, respectively, which are similar to the kinetic parameters of L-dopa (Table 2). A similar *K*_*m*_ value of 0.25 mM was reported by García-Canovas and co-workers for *Agaricus bisporus* tyrosinase^26^.

### Effects of KA and HQ on the kinetic parameters of TyrBm

The kinetic constants of TyrBm monophenolase and diphenolase activities were determined for L-tyrosine and L-dopa (Table 2). The values obtained in the presence of KA and HQ were calculated from Lineweaver–Burk plots (Figs 1 and 2; Table 1). With rising concentrations of KA, the *K*_m_ values of the monophenolase activity increased and the *V*_max_ values decreased, an indication of a mixed mode of inhibition, with an inhibition constant *K*_I_ of 1.1 μM and *K*_IS_ of 61 μM. The apparent *K*_m_ and *V*_max_ were 0.04 mM and 9.7 μmole min^−1^ mg^−1^, respectively (Table 1). When increasing concentrations of KA were added in the presence of L-dopa as the substrate, a similar mode of mixed inhibition was observed, with *K*_I_ of 3.5 μM and *K*_IS_ of 150 μM, similar to previous studies that reported *K*_I_ values of 3.4, 5 and 4.7 μM for mushroom tyrosinase^18^,^19^,^33^. The apparent *K*_m_ and *V*_max_ of the diphenolase reaction were 0.18 mM and 34 μmole min^−1^ mg^−1^, respectively (Table 1). The mixed inhibition mode implies that KA binding is not limited to the active site. In a previous study we have already experimentally demonstrated that a peripheral KA binding site exists in TyrBm^22^.

Increasing concentrations of HQ in the presence of L-tyrosine, resulted in an increase in the *K*_m_ value while the *V*_max_ remained constant, an indication of a competitive inhibition mode on the monophenolase activity, with a *K*_I_ of 40 μM and apparent *K*_m_ and *V*_max_ values of 0.07 mM and 9.0 μmole min^−1^ mg^−1^, respectively (Fig. 2a and Table 1). A similar inhibition mechanism was also reported by Chawla *et al.*, with *K*_I_ of 83 μM for mushroom tyrosinase^25^.

### Binding affinity of L-tyrosine vs. inhibitors

In order to obtain a clearer understanding of the fashion by which substrates and inhibitors bind to tyrosinase, we have determined dissociation constants (*K*_D_) between TyrBm and its substrates or inhibitors using MicroScale Thermophoresis^34^. Surprisingly, such *K*_D_ values had not been previously measured. These experiments were performed by titrating fluorescently-labeled TyrBm with increasing concentrations of KA, HQ or L-tyrosine as the unlabeled ligands. According to the thermophoretic data points obtained with increasing concentrations of the ligands, the dissociation constants were evaluated. The *K*_D_ values of the TyrBm-KA, TyrBm-HQ and TyrBm-L-tyrosine interactions were determined as 377, 9 and 0.1 μM, respectively (Table 3 and Supplementary Fig. S4). According to these results, L-tyrosine, the natural substrate, showed the highest affinity to TyrBm in comparison with KA and HQ. While HQ showed a dissociation constant 90-fold higher than L-tyrosine, KA exhibited a value of nearly 4000-fold higher than the natural substrate.

### Structure of TyrBm with KA in the active site

**Figure 3:**
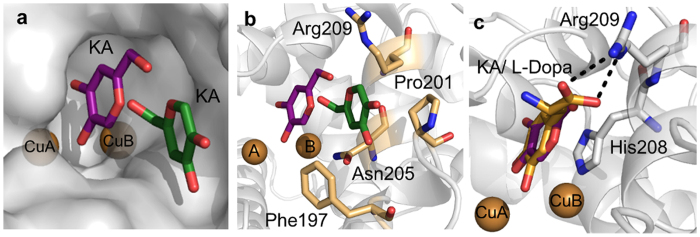
Structures of KA bound to TyrBm. (**a**) KA is observed inside the active site (purple) and at the entrance to the active site (green) (3NQ1). Copper ions are presented as brown spheres. (**b**) KA at the entrance to the active site (green) (3NQ1) is stabilized by second shell residues (light brown sticks). (**c**) Superposition with TyrBm structures contain KA (purple) and L-dopa (orange, 4P6S) oriented through hydrophobic interactions with His208. All the structures presented in this work were generated using PyMOL.

In addition to the peripheral binding site of KA (PDB 3NQ1), we have recently determined the structures of TyrBm with L-tyrosine, L-dopa and the substrate analog *p*-tyrosol^35^, all found within the active site. We present here the crystal structure of TyrBm with KA bound in the active site at 2.6 Å resolution (Fig. 3, Supplementary Fig. S5 and Table 4). The possible movement of KA within the active site can be envisioned from Fig. 3a in which KA is shown in two positions: the peripheral site we reported earlier^22^, and in the active site. At the entrance to the active site, KA is stabilized by interactions with Phe197, Pro201, Asn205, and Arg209 (Fig. 3b)^22^. In the active site, KA is stabilized by π-π interactions with His208 that coordinates CuB, similar to tyrosinase substrates (Fig. 3c), as presented by Goldfeder *et al.* and suggested in other studies^35^,^36^,^37^. The hydroxyl group of KA is oriented towards CuA with a distance of 3.3 Å, while the distance of the carbonyl group to CuA is 5.5 Å. These results are supported by a recent docking study of Lima *et al.*^18^, and contradict a previously proposed inhibition mechanism of KA by copper chelation^20^,^21^,^22^.

### *In silico* simulations of KA and HQ in the active site

**Figure 4:**
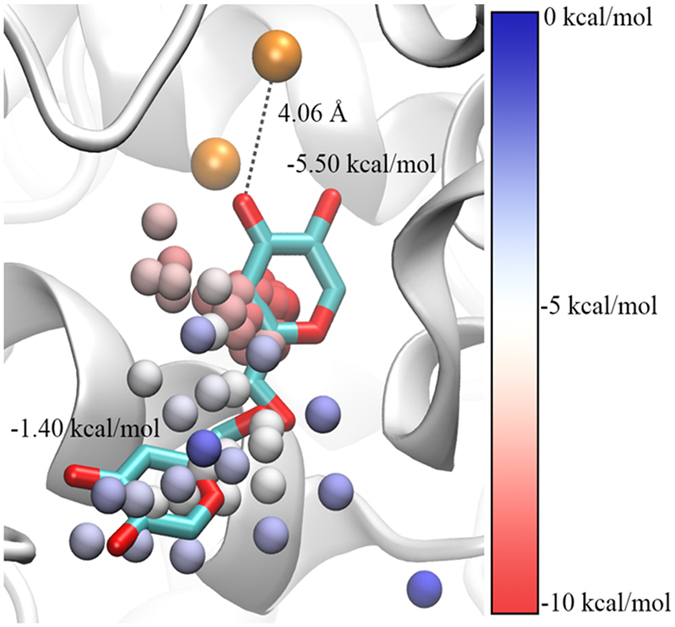
KA’s center of mass cluster analysis along the PELE simulation. Clusters are presented as spheres and colors indicate the potential of mean field ∆G. Absolute standard binding free energies (with volume corrections) are shown for the active site and the surface bound minima, along with the ligand crystallographic complexes (cyan sticks). The two copper ions are presented as brown spheres.

TyrBm structure with KA at the entrance to the active site was used as an initial model to run an extensive non-biased ligand migration simulations with PELE (Protein Energy Landscape Exploration) in a constrained sphere of 20 Å (from the initial ligand center of mass)^38^. By means of 128 processors and 24 hours, ~200,000 different ligand conformations were obtained that allowed to evaluate the absolute binding free energy (∆G) by Markov State Models (MSM) analysis^39^. Briefly, MSM first involves clustering all conformations (a total number of 100 clusters were used) in metastable states and building the transition matrix between them. The obtained clusters overlap mostly with the two positions of KA, at the peripheral site and in the active site (Fig. 4). Integration of these cluster centers (with respect to the bulk solvent) allowed determining the binding free energy for the active site structure of −5.5 kcal/mol, whereas the surface bound complex was only of −1.4 kcal/mol. Therefore, the transition from the surface bound complex to the active site is exothermic and likely to occur.

An analogous simulation was also performed for HQ. In contrast to KA, HQ showed a significant larger mobility in the active site, where multiple orientations are frequently visited. This is clearly seen when analyzing the metastable states (after MSM clustering) accessible within 1 kcal/mol from the best-bound minima (Supplementary Fig. S6). While KA presents mainly two orientations (that occupy similar volume), HQ adopts multiple orientations exploring a larger area of the active site. Interestingly, for HQ we found structures (within the lowest 1 kcal/mol) involving the peripheral site, which for KA is about 4.1 kcal/mol above the best-bound minima (Fig. 4).

### Structure of TyrBm with HQ in the active site

**Figure 5:**
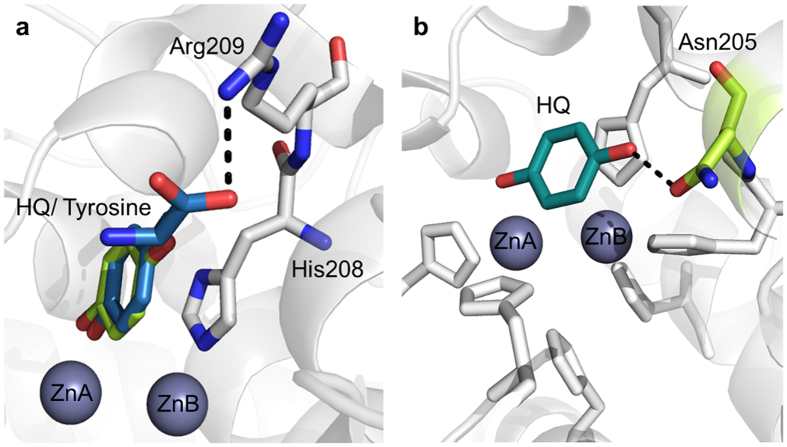
Structures of HQ bound in the active site of TyrBm. (**a**) Superposition with TyrBm structures contain HQ in orientation 2 (green) and L-tyrosine (blue, 4P6R), which forms a hydrogen bond with Arg209. Zinc ions are presented as grey spheres. (**b**) HQ, in orientation 1 (teal), forms a hydrogen bond with Asn205 His residues are in white.

The kinetic measurements with HQ indicated that it is a poor substrate of TyrBm under natural conditions, and a good substrate in conditions favoring *oxy*-tyrosinase. In order to trap HQ in the active site of TyrBm, mature crystals were soaked with zinc instead of copper ions to prevent enzymatic activity^22^,^35^,^40^. We have obtained two different structures of TyrBm with HQ bound in the active site (Supplementary Figs S7 and S8) at 2.2 Å resolution (Table 4). The HQ hydroxyl group is oriented towards ZnA, and its benzyl ring is stabilized through hydrophobic interactions with His208, similar to tyrosine substrates (Fig. 5a)^35^. HQ was observed to bind in three different orientations in total in the active site of TyrBm (orientations 1, 2 and 3) (Fig. 5, Supplementary Figs S7 and S8). It seems that HQ binding is rather flexible in the active site, agreeing with the *in silico* simulations shown above, and does not have one specific orientation in contrast to L-tyrosine and L-dopa (Fig. 5)^35^. In orientation 1, a polar interaction between HQ and Asn205 is observed (Fig. 5b). In orientation 2, the HQ molecule is oriented similarly to tyrosinase substrates (and KA) in the active site, supporting our kinetic experiments showing that HQ can act as a TyrBm substrate (Fig. 5a). In addition, when TyrBm crystals were soaked with copper and HQ for 16 hours, the crystals turned brown, in comparison to crystals that were soaked with zinc that did not show a change in color (data not shown). Brown TyrBm crystals indicate on substrate oxidation as was previously shown by Sendovski *et al.* and provide additional confirmation on the role of HQ as a substrate of TyrBm^22^.

## Discussion

Disorders in melanin formation have been linked to various skin diseases in humans such as hyperpigmentation and skin cancer. KA and HQ, are frequently used as inhibitors of tyrosinase, and have been used as skin-whitening agents in leading cosmetic hyperpigmentation treatment^9^,^13^,^14^,^16^. Over the past few years, numerous docking studies and molecular dynamic simulations were performed in an attempt to elucidate the binding modes of tyrosinase inhibitors. In this work we demonstrate for the first time the true binding orientations of KA and HQ in the active site of TyrBm which explain the biochemical characterization.

Previously, we had determined a crystal structure of TyrBm with KA bound at the entrance to the active site^22^. Here, by modifying our protocol for ligand binding *in crystal*, we have visualized the structure of TyrBm with KA bound in the active site similar to tyrosinase substrates (Fig. 3c). This position of KA might lead to the false assumption of competitive inhibition mechanism. However, the two orientations of KA, which are demonstrated by crystallography and *in silico* simulations (Figs 3 and 4), support the mixed inhibition mechanism, which is confirmed by our kinetic experiments (Fig. 1 and Table 1).

In contrast to previous studies^18^,^19^,^20^, in this work we unequivocally display mixed inhibition mode of KA on both monophenolase and diphenolase activities and undermine the hypothesis regarding copper chelation by KA. Since the *K*_IS_ value is significantly greater than *K*_I_ for the oxidation of both L-tyrosine and L-dopa (Table 1), KA is able to bind more strongly to the free enzyme than to the enzyme–substrate complex (at the peripheral site). We suggest that when KA is bound strongly in the active site, the binding pocket is not accessible to substrate molecules, subsequently TyrBm is not active. However, when KA is oriented at the entrance of the active site, it restricts substrate entrance and product efflux, consequently, TyrBm cannot reach its maximum velocity (Table 1). Tropolone, another tyrosinase inhibitor that has been studied, was also found at the entrance of the active site of mushroom tyrosinase and exhibited mixed inhibition mode similar to KA^41^,^42^. It is quite possible, that KA may also bind to this site or has a different peripheral binding site yet to be elucidated. Together with the fact that KA showed a dissociation constant 3-orders of magnitude higher than L-tyrosine, these findings support the existence of a significant intermediate binding site in TyrBm and explains the mechanism of mixed inhibition.

Numerous studies have raised questions regarding the behavior of HQ as a tyrosinase inhibitor^26^,^27^, and most of them characterized HQ as a competitive inhibitor^24^,^43^,^44^,^45^. Our results support this inhibition mode of HQ on L-tyrosine (Fig. 2a and Table 1). On the other hand, other studies demonstrated the potency of HQ as a tyrosinase substrate^26^,^27^. Stratford and co-workers suggested that HQ is neither a substrate nor an inhibitor of tyrosinase^27^ while del Mar García-Molina *et al.* suggested that HQ is a tyrosinase substrate with a poor activity due to the inability to transform *met*-tyrosinase to *oxy*-tyrosinase on its own^26^. The transformation is achieved by addition of an *o*-diphenol (such as L-dopa) or H_2_O_2_, which promotes the activity on HQ^26^,^28^. Our results corroborate this argument, since in the presence of H_2_O_2_ or L-dopa, TyrBm was indeed active on HQ as measured by two unrelated methods (Fig. 2b and Supplementary Figs S1 and S3). These findings led us to determine the kinetic constants of TyrBm with HQ as a substrate, which resulted in a similar *K*_m_ value for HQ and L-dopa, and a *V*_max_ value higher than that of L-tyrosine (Table 2).

Additional conclusive evidence for the action of TyrBm on HQ lies in the formation of brown crystals soaked in HQ that indicate on substrate oxidation as was previously shown by Sendovski *et al.* with L-tyrosine^22^. We assume that small amounts of *oxy*-TyrBm molecules present in the crystals enabled the activity on HQ within 16 hours that resulted in brown pigmentation^9^.

In order to elucidate the inhibition mechanism of HQ, we solved two crystal structures of TyrBm with HQ in the active site. It was discovered that HQ is bound less strongly than L-tyrosine (Table 3), and its binding heterogeneity is evident from the several different orientations observed in the active site (Fig. 5 and Supplementary Fig. S7). It is presumed that the polar amine and carboxyl groups of L-tyrosine and L-dopa, which are not present in HQ, help to stabilize the substrates through polar interactions with Arg209 in a productive mode (Fig. 5a)^35^. In orientation 1, a hydrogen bond between HQ and Asn205 was observed (Fig. 5b). Asn205 was suggested to be crucial for tyrosinase activity through the activation of a conserved water molecule^35^,^46^. The interaction of Asn205 with HQ might prevent this activation, and thus inhibit tyrosinase activity^18^,^47^,^48^. Furthermore, in the structures of TyrBm with KA at the entrance of the active site this interaction was also found to be important for KA stabilization^22^. Thus, we propose that the polar bond between Asn205 and the hydroxyl group of HQ indicates on an inhibitory effect on TyrBm. In contrast, in orientation 2, HQ is positioned similarly to L-tyrosine in the active site (Fig. 5a), supporting the role of HQ as TyrBm substrate. The flexibility of HQ in the active site of TyrBm was also demonstrated by our PELE simulations, which provided visualization of the numerous energetically feasible orientations of HQ in the active site. Whereas for KA it takes some time and energy to go from the peripheral docking site to the inner active site, for HQ there is constant interconversion between the two of them suggesting that the pre-docking site is very transient.

It seems that the combination of both the orientation of HQ in the active site and the oxidative state of tyrosinase will define the behavior of HQ.

## Methods

### Expression, purification and crystallization of tyrosinase from *B. megaterium*

The gene encoding tyrosinase from *Bacillus megaterium* (TyrBm) was cloned into *Escherichia coli* BL21, purified and crystallized as previously described^49^,^50^.

### Tyrosinase inhibition assay

Tyrosinase inhibitory activity was determined spectrophotometrically in 96-well plates with a final volume of 200 μl. First, 50 mM PBS buffer pH 7.4 and 0.01 mM CuSO_4_ were mixed with 6 μg ml^−1^ of purified enzyme. Then, the mixture was incubated at 40 °C for 2 minutes. Finally, various concentrations of inhibitor were mixed with 1.2 mM L-tyrosine or 2 mM L-dopa and were added to the pre-incubated mixture. KA was studied in the range of 0.025–0.1 mM and HQ in the range of 0.025–0.5 mM. The reaction mixture was then monitored for L-dopachrome formation (ε = 3600M^−1^ cm^−1^) by measuring the absorbance at 475 nm. Specific activity was calculated as the ratio of the conversion rate and the total protein content as determined by the Bradford analysis method (Bio-Rad, Israel). All measurements were performed in seven replicates. The inhibitor concentration necessary for 50% inhibition (IC_50_) was determined with respect to a control (no inhibitor).

### Kinetic analysis of tyrosinase

The mode of inhibition and inhibition parameters, i.e. the Michaelis–Menten constant (*K*_m_), maximal velocity (V_max_), turnover number (*k*_cat_) of TyrBm and the inhibition constants (*K*_I,_ *K*_IS_) of each inhibitor were determined by Lineweaver–Burk plot analysis using various concentrations of L-tyrosine (0.03–1.4 mM) and L-dopa (0.15–2.0 mM) as substrates. The inhibitor concentrations were mentioned above. The inhibition kinetics module of Sigma Plot 13.0 software was used (Systat Software, Inc., Richmond, CA, USA). All measurements were performed in 5-replicates.

### Tyrosinase activity assay on HQ as a substrate

TyrBm activity was determined by measuring the formation of 2-hydroxy-*p*-benzoquinone (HPB) from HQ, in the presence of H_2_O_2_ or L-dopa. TyrBm activity was determined as explained in the inhibition assay above with varying concentrations of H_2_O_2_, ranging from 0 to 90 mM, while maintaining the concentration of HQ constant. The formation of HBP was monitored by measuring the absorbance at 475 nm^26^.

The values of *K*_m,_ V_max_ and *k*_cat_ of TyrBm in the presence of HQ as a substrate were determined with the following conditions: 50 mM PBS buffer pH 7.4, 0.01 mM CuSO_4_, 6 μg ml^−1^ of purified enzyme, various concentrations of HQ (0.1–2.0 mM) in the presence of saturating concentration of hydrogen peroxide (100 mM)^51^.

For further verification of TyrBm activity on HQ, the activity was determined by recording the oxygen consumption in the presence of L-dopa and HQ. Measurements were carried out using a Hansatech Oxygraph+ electrode (Norfolk, UK) in a reaction volume of 1000 μL. The reaction contained 4 μg ml^−1^ of purified TyrBm, 50 mM PBS buffer pH 7.4, 0.01 mM CuSO_4_, 1 mM L-dopa and 0.1 mM HQ.

### Dissociation constants using MicroScale Thermophoresis (MST)

TyrBm was labeled fluorescently with a RED dye (NT-647-NHS) according to the manufacturer’s protocol (NanoTemper Technologies, Munich, Germany). Non-bound dye was removed by purification of the enzyme on a Sephadex G-25 column with buffers provided in the commercial kit. Then, serial dilutions of unlabeled binding partner samples (inhibitor or substrate) were mixed with 0.377 μM of dye-labeled TyrBm in 50 mM PBS buffer pH 7.4 and incubated for 5 minutes. Approximately 10 μl of sample was loaded into hydrophilic monolith NT capillaries and the measurement was performed in a NanoTemper Monolith NT.015T instrument. The emission of the red fluorescence was recorded at a focused location of the capillary. In the same location, a microscopic temperature gradient was created using an infrared laser and the fluorescence depletion was measured. According to changes in the fluorescent thermophoresis signal and the concentrations of unlabeled inhibitor, the dissociation constant values were determined by the NanoTemper analysis software. The unlabeled binding partners tested for *K*_D_ determination were KA (0–4 mM), HQ (0–1 mM) and L-tyrosine (0–2 mM).

### Statistical analysis

All experiments were performed in duplicates or triplicates in order to ensure the reproducibility of the results. Statistical analysis was performed using Student’s t-test: *P < 0.05 compared with the control. Data is summarized as mean ± SD.

### Substrate binding in crystals

In order to trap ligands in the active site, mature crystals were soaked overnight in 1 mM of either CuSO_4_ or ZnCl_2_ and subsequently in 10 mM of the appropriate ligand (KA and HQ) before crystal freezing.

### Data collection and structure determination

X-ray diffraction data was collected at the European Synchrotron Radiation Facility, Grenoble, France, at beamlines ID14-4 and ID 29. All data were indexed, integrated, scaled and merged using Mosflm and Scala^52^. The structures of TyrBm with bound inhibitors were solved by molecular replacement using Phaser^53^ and the coordinates of earlier determined TyrBm structure (PDB code 4P6R). Refinement was performed using Phenix^54^ and Refmac5^55^,^56^, coupled with rounds of manual model building, real-space refinement and structure validation performed using COOT^57^. Data collection, phasing and refinement statistics are presented in Table 3.

### *In silico* simulations

Ligand migration sampling with Protein Energy Landscape Energy (PELE). PELE has widely been used to study ligand-protein interactions and protein dynamics at a fraction of the cost compared to other sampling methods such as molecular dynamics^58^,^59^,^60^. This algorithm is composed of a perturbation and a relaxation stage, and uses a mixture of random moves with protein structure prediction algorithms. The resulting structure is accepted or rejected following the Metropolis criterion.

Binding free energy with Markov State Models (MSM). MSM are coarse grain statistical models that allow extracting equilibrium properties such as the binding free energy^61^. In order to build our MSM, we split the conformational space using the Voronoi decomposition, clustering the ligand’s center of mass and using the cluster centers as seeds. Hence, each microstate will contain all possible ligand, protein and solvent arrangements compatible with having the ligand’s center of mass within the cell. In order to study the different metastable minima, microstates are kinetically clustered utilizing Perron Cluster Analysis (PCCA+). The absolute binding free energy, ∆G, is obtained integrating the potential of mean force (Gpmf) in the whole bound region^39^.

## Additional Information

**Accession codes**: The coordinates and structure factors of TyrBm in different states have been deposited in the RCSB PDB under accession codes 5I3B (TyrBm with configuration B of HQ), 5I3A (TyrBm with configuration A of HQ) and 5I38 (TyrBm with KA).

**How to cite this article**: Deri, B. *et al.* The unravelling of the complex pattern of tyrosinase inhibition. *Sci. Rep.* **6**, 34993; doi: 10.1038/srep34993 (2016).

## Supplementary Material

Supplementary Information

## Figures and Tables

**Table 1:** Kinetic and inhibition constants of TyrBm by KA and HQ.

	Substrate	*K*_m_ (mM)	*V*_max_ (μmole min^−1^ mg^−1^)	*k*_cat_ (s^−1^)	IC_50_ (μM)	*K*_I_ (μM)	*K*_IS_ (μM)	Inhibition mode
KA	L-tyrosine	0.04 ± 0.007	9.7 ± 0.4	5.7	26.8 ± 0.8	1.1 ± 0. 3	61 ± 20	mixed
	L-dopa	0.18 ± 0.03	34 ± 1	20.1	52±3	3.5 ± 0. 6	150 ± 12	mixed
HQ	L-tyrosine	0.07 ± 0.01	9.0 ± 0.5	5.3	32 ± 2	40 ± 10	—	competitive

Data was extracted from Figs 1, 2 and Supplementary Fig. S1. Each value represents the mean ± SD of five independent experiments.

**Table 2:** Kinetic constants of TyrBm on its natural substrates and HQ.

Substrate	*K*_m_ (mM)	*V*_max_ (μmole min^−1^ mg^−1^)	*k*_cat_ (s^−1^)	*k*_cat_*/K*_m_ (s^−1^ mM^−1^)
L-tyrosine	0.082 ± 0.006	3.62 ± 0.06	2.1	25.6
L-dopa	0.24 ± 0.02	30.3 ± 0.6	17.8	74.2
HQ	0.27 ± 0.05	19 ± 1	11.3	41.9

Data was extracted from Figs 1, 2 and Supplementary Fig. S1. Each value represents the mean ± SD of five independent experiments.

**Table 3:** Dissociation constants of TyrBm-ligand complexes.

TyrBm ligand	*K*_D_ (μM)
KA	377 ± 4
HQ	9 ± 1
L-tyrosine	0.10 ± 0.03

Each value represents the mean ± SD.

**Table 4:** Data collection and refinement statistics.

Structure name (PDB code)	TyrBm:KA (5I38)	TyrBm:HQA (5I3A)	TyrBm:HQB (5I3B)
*Data collection*			
Space group	P2_1_2_1_2_1_	P2_1_2_1_2_1_	P2_1_2_1_2_1_
Cell dimensions			
*a, b, c* (Å)	70.24, 74.97, 121.70	69.62, 74.38, 120.78	69.62, 74.42, 119.69
*α, β, γ (°)*	90, 90, 90	90, 90, 90	90, 90, 90
Resolution (Å)	51.26–2.5	35.54–2.2	33.43–2.2
*R*merge*,†	0.12(0.25)	0.082(0.387)	0.08(0.326)
I/σI*	9.7(5.9)	18.2(6.2)	15.4(5.8)
Completeness*	90.4(99.3)	99.9(100)	99.8(99.9)
Redundancy*	6.2(5.8)	12.6(13.1)	6.6(6.9)
Refinement			
Resolution (Å)	51.26–2.5	35.54–2.2	33.43–2.2
No. of reflections	127,679	558,159	214,516
*R*work/*R*free‡	20.81/23.77	20.11/22.72	18.84/21.69
No. of atoms			
Protein	4,687	4,697	4,696
Ligand/ion	36	33	33
Water	84	226	272
B-factors (Å)			
Protein	32.05	35.02	32.75
Ligand/ion	33.51	39.86	29.39
Water	30.74	37.73	35.42
Root mean square deviations			
Bond length (Å)	0.004	0.007	0.006
Bond angle (°)	0.59	0.88	0.80

^*^Values in parentheses are for the last shell.

^†^*R*_merge_ = ∑_hkl_∑_*i*_ |I_*i*_(hkl)−〈I(hkl)〉|/∑_hkl_∑_*i*_I_*i*_(hkl), where *I* is the observed intensity, and <*I*> is the mean value of *I*.

^‡^*R/R*_free_ = ∑_hkl_||F_obs_|−|F_calc_||/∑_hkl_|F_obs_| where *R* and *R*_free_ are calculated using the test reflections respectively. The test reflections (5%) were held aside and not used during the entire refinement process.
