# Supplementary material for: The unravelling of the complex pattern of tyrosinase inhibition
Source: Sci Rep. 2016 Oct 11;6:34993. doi: 10.1038/srep34993 (PMC5057104; doi:10.1038/srep34993)
Supplement: Supplementary Information [file srep34993-s1.pdf]

Supplementary Data

The unravelling of the complex pattern of tyrosinase inhibition

Batel Deri[a,§], Margarita Kanteev[a,§], Mor Goldfeder[a], Daniel Lecina[b], Victor Guallar[b,c], Noam Adir[d], and Ayelet Fishman[a,†]

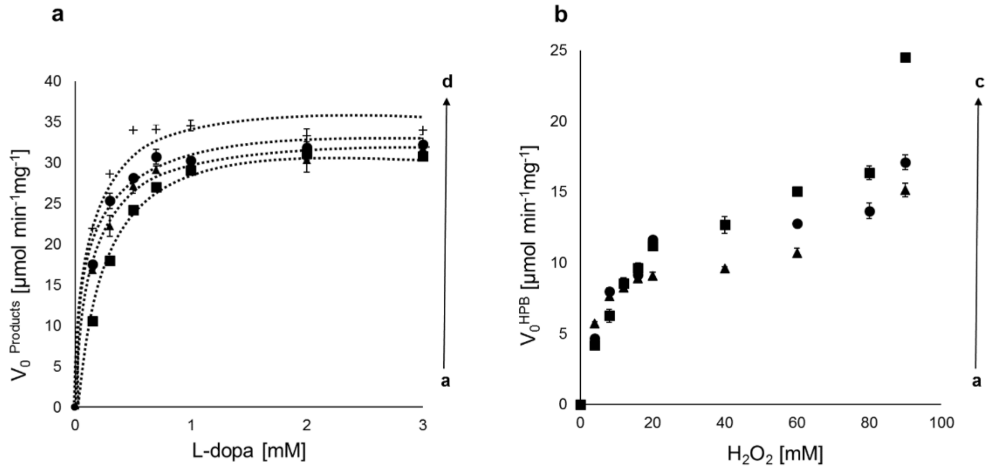

Supplementary Figure S1: Initial product formation rate of TyrBm activity on HQ in the presence of reducing agents measured at 475 nm. (a) HQ as the substrate in the presence of increasing concentrations of L-dopa (0-3 mM). HQ concentrations (mM) were: (a ■) 0, (b ♦) 0.025, (c ●) 0.075 and (d +) 0.5. The product may be composed of HPB (2-hydroxy-*p*-benzoquinone) and dopa-quinone since both HQ and L-dopa may serve as substrates. (b) HQ as the substrate in the presence of increasing concentrations of H_2O_2 (0-90 mM). HQ concentrations (mM) were: (a ▲) 0.1, (b ●) 0.5 and (c ■) 1.5. The product is HPB only. The reactions contained 6 µg ml^{-1} of purified TyrBm, 50 mM PBS buffer pH 7.4 and 0.01 mM $CuSO_4$.

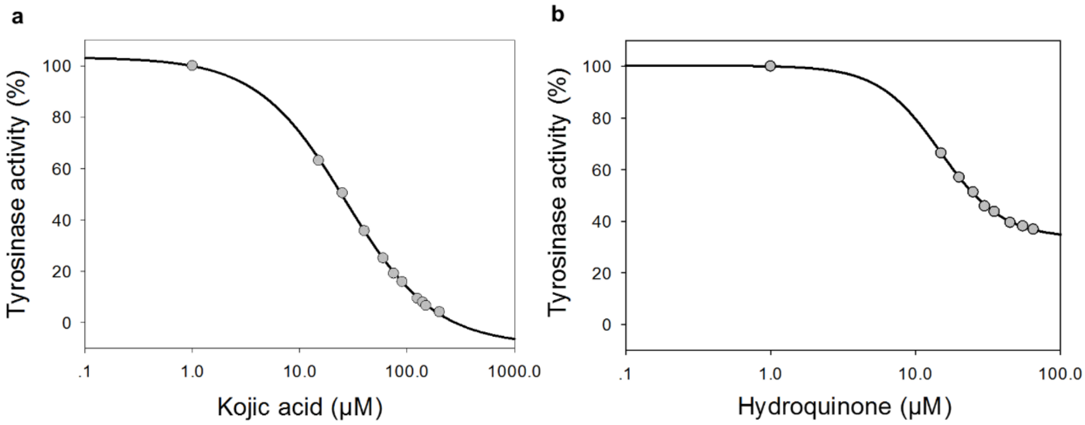

Supplementary Figure S2: Inhibition of TyrBm monophenolase activity by (a) KA and (b) HQ. IC50 values were determined at 50% activity.

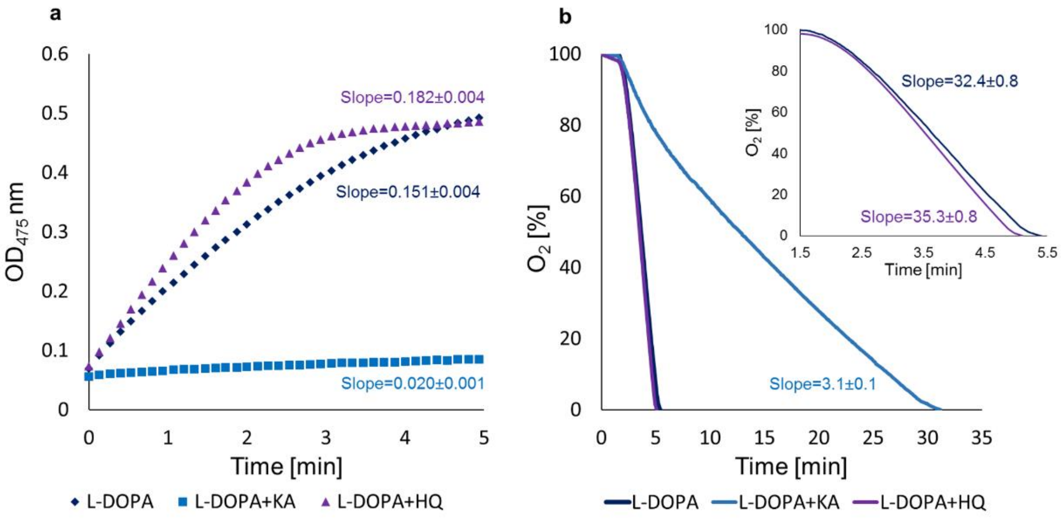

Supplementary Figure S3: Action of TyrBm on L-dopa alone or in the presence of KA and HQ. (a) Spectrophotometric recordings measured at 475 nm. All measurements were performed in heptaplicates. (b) Oxygen consumption recordings. The inset presents the oxygen consumption in the presence of L-dopa and L-dopa with HQ in the first 5 minutes. All measurements were performed in triplicates. The reactions contained 4 µg ml^{-1} of purified TyrBm, 50 mM PBS buffer pH 7.4, 0.01 mM $CuSO_4$, 1 mM L-dopa, 0.1 mM HQ and 0.1 mM KA. The slopes representing the activity rate are given for each graph. KA clearly inhibits TyrBm activity, whereas HQ enhances activity.

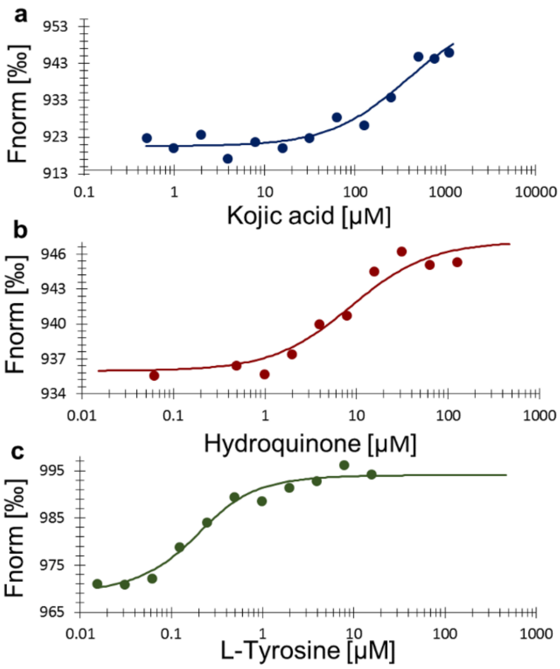

Supplementary Figure S4: Microscale thermophoresis (MST) analysis showing the binding of TyrBm-ligand by change in fluorescence. (a) Titration of rising concentrations of KA (0-4 mM) induces MST signal and yields K_D of 377±4 µM. (b) Titration of rising concentrations of HQ (0-1mM) yields K_D of 9±1 µM. (c) Titration of rising concentrations of L-tyrosine (0-2mM) yields K_D of 0.10±0.03 µM. The reactions contained constant concentration of TyrBm (0.377 µM) in 50 mM PBS buffer pH 7.4.

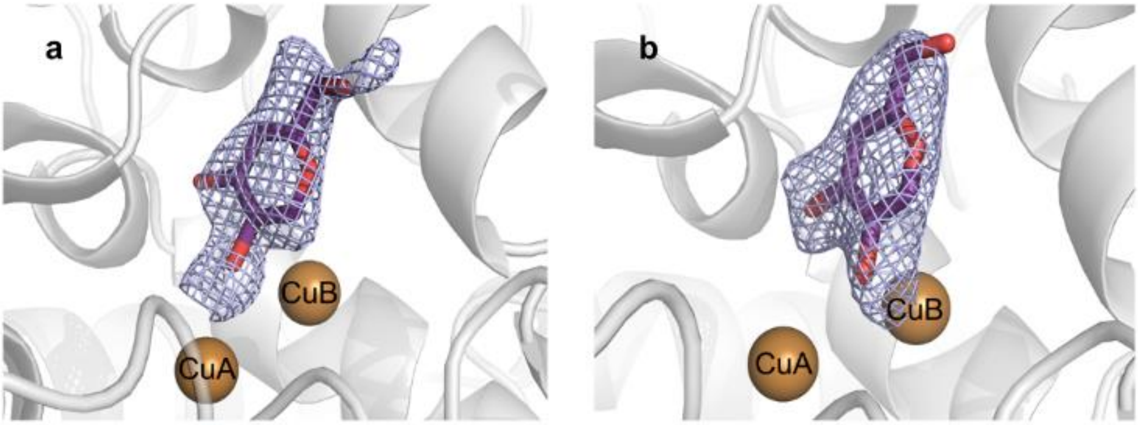

Supplementary Figure S5: KA with its mF_O-DF_C electron density omit map (light blue wire) contoured at 2σ. (a) KA in the active site of monomer A, structure 5I38. (b) KA in the active site of monomer B, structure 5I38. Copper ions are presented as brown spheres.

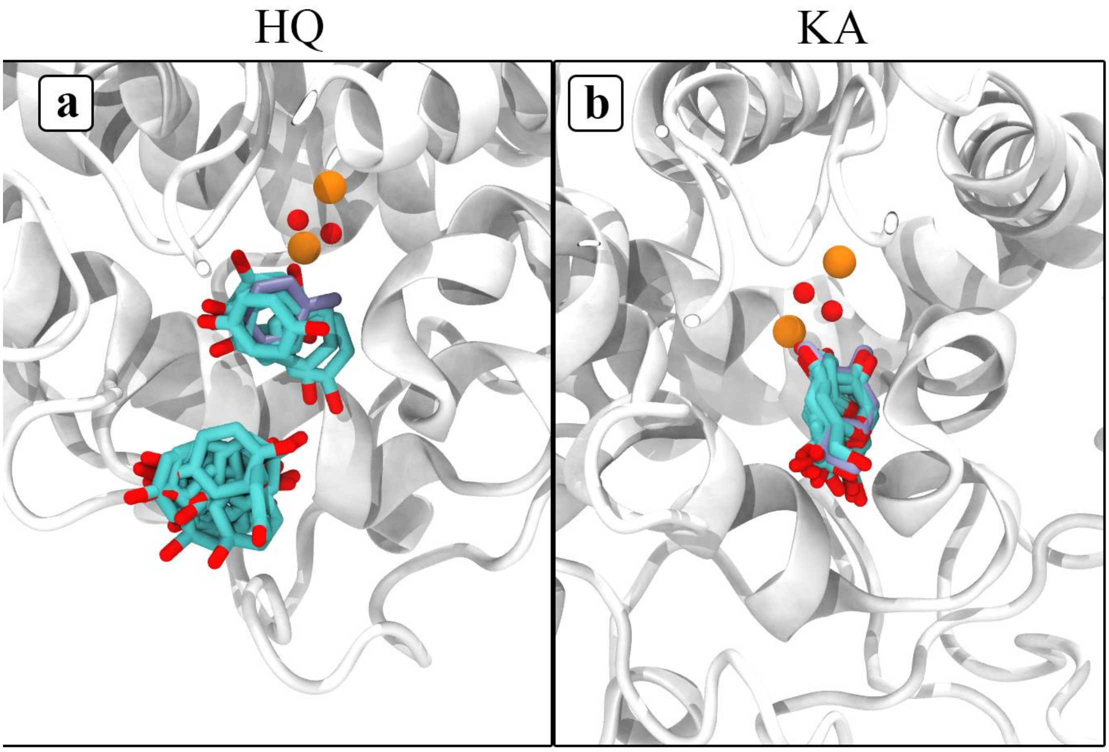

Supplementary Figure S6: Representative structures from the MSM clusters for HQ (panel a) and KA (panel b) within 1 kcal/mol of the best bound complex (highlighted in darker blue). Copper ions are presented as brown spheres, water molecules in red.

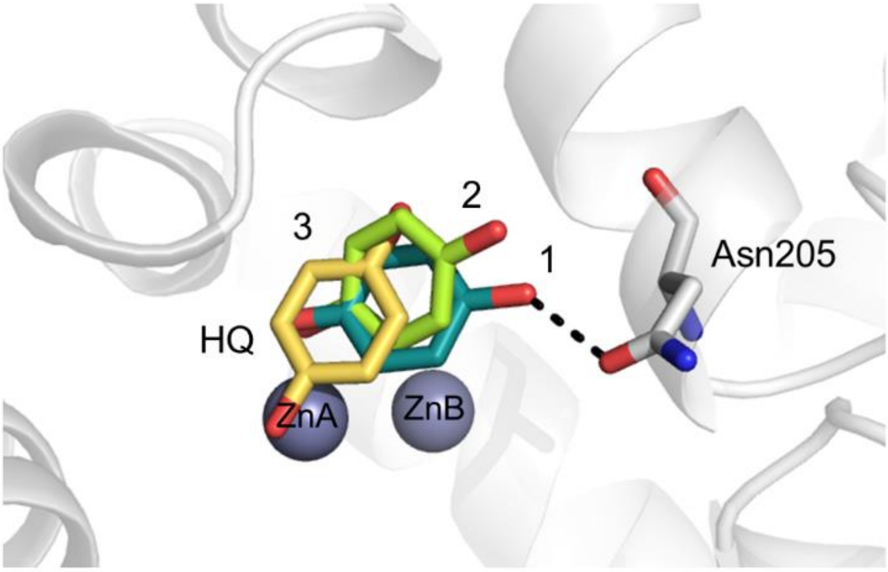

Supplementary Figure S7: Positions of HQ in the active site of TyrBm. Superposition of the active site of TyrBm monomers, obtained from two crystal structures with HQ in the active site. In orientation 1 of HQ (deep teal), the hydroxyl side chain forms a hydrogen bond with Asn205 (monomer A, 5I3B). In orientation 2 (green), HQ is oriented similar to tyrosinase substrates (monomer B, 5I3B). The different orientations of HQ (1, 2 and 3) represent flexibility in the active site. Zinc ions are presented as grey spheres. HQ in the active site of monomer A, 5I3A, is positioned in orientation 1 as well (data not shown).

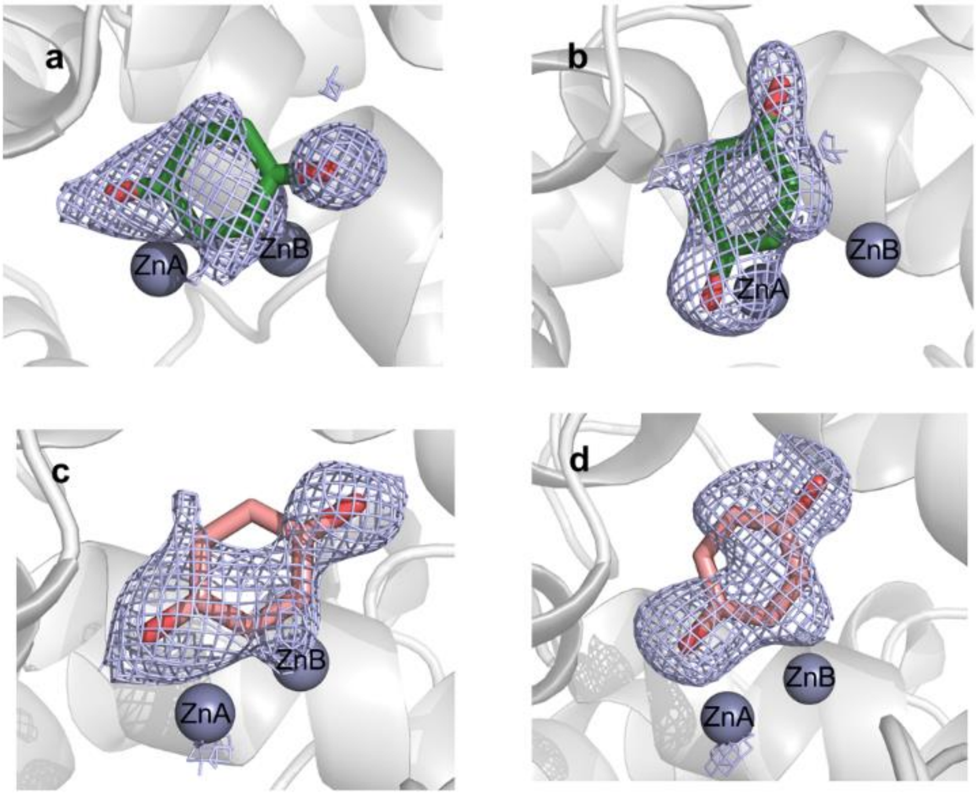

Supplementary Figure S8: HQ with its mF_O-DF_C electron density omit map (light blue wire) contoured at 2σ. (a) HQ (in orientation 1) in the active site of monomer A, structure 5I3A. (b) HQ (in orientation 2) in the active site of monomer B, structure 5I3A. (c) HQ (in orientation 1) in the active site of monomer A, structure 5I3B and (d) HQ (in orientation 3) in the active site of monomer B, structure 5I3B. Zinc ions are presented as grey spheres.
